# Observers’ Agreement on Measurements in Fiberoptic Endoscopic Evaluation of Swallowing

**DOI:** 10.1007/s00455-015-9673-7

**Published:** 2016-01-23

**Authors:** Walmari Pilz, Sophie Vanbelle, Bernd Kremer, Michel R. van Hooren, Tine van Becelaere, Nel Roodenburg, Laura W. J. Baijens

**Affiliations:** Department of Otorhinolaryngology, Head and Neck Surgery, Maastricht University Medical Center, P.O. Box 5800, 6202 AZ Maastricht, The Netherlands; Department of Methodology and Statistics, Maastricht University, Maastricht, The Netherlands; Department of Neurology, Maastricht University Medical Center, Maastricht, The Netherlands

**Keywords:** Deglutition, Deglutition disorder, Observer agreement, Fiberoptic endoscopic evaluation of swallowing (FEES)

## Abstract

This study analyzed the effect that dysphagia etiology, different observers, and bolus consistency might have on the level of agreement for measurements in FEES images reached by independent versus consensus panel rating. Sixty patients were included and divided into two groups according to dysphagia etiology: neurological or head and neck oncological. All patients underwent standardized FEES examination using thin and thick liquid consistencies. Two observers scored the same exams, first independently and then in a consensus panel. Four ordinal FEES variables were analyzed. Statistical analysis was performed using a linear weighted kappa coefficient and Bayesian multilevel model. Intra- and interobserver agreement on FEES measurements ranged from 0.76 to 0.93 and from 0.61 to 0.88, respectively. Dysphagia etiology did not influence observers’ agreement level. However, bolus consistency resulted in decreased interobserver agreement for all measured FEES variables during thin liquid swallows. When rating on the consensus panel, the observers deviated considerably from the scores they had previously given on the independent rating task. Observer agreement on measurements in FEES exams was influenced by bolus consistency, not by dysphagia etiology. Therefore, observer agreement on FEES measurements should be analyzed by taking bolus consistency into account, as it might affect the interpretation of the outcome. Identifying factors that might influence agreement levels could lead to better understanding of the rating process and assist in developing a more precise measurement scale that would ensure higher levels of observer agreement for measurements in FEES exams.

## Introduction

Fiberoptic endoscopic evaluation of swallowing (FEES) has been widely used to evaluate oropharyngeal dysphagia since it was first described in 1988 [1]. Besides being safe and easy to use, FEES permits the anatomical assessment of the pharyngeal and laryngeal structures; it also constitutes a comprehensive evaluation of the pharyngeal stage of swallowing [2]. For these reasons, both diagnosis and treatment planning of deglutition disorders often take FEES outcome measurements into account. While the popularity of FEES as an assessment tool is increasing, research on standardization and validation of measurement criteria in these exams lags behind. Crucially, interpretation of swallowing images is based on visual judgment and is thus subjective. It might be influenced by factors such as experience of the observer(s), bolus consistency, and dysphagia severity [3–5]. Moreover, the literature on swallowing evaluation rarely describes the protocols or the variables analyzed in sufficient detail [6]. A few studies have addressed observer agreement on some well-known visuoperceptual ordinal variables, such as the Penetration Aspiration Scale (PAS) and the pharyngeal residue scale. Nonetheless, the variability in the scoring of FEES exams remains underexplored [7–10]. Given its role in clinical decision making, an accurate and reliable measurement technique is necessary.

In this paper, rather than simply report estimate agreement indexes, a statistical multilevel approach method was used to analyze the data. This method quantifies the impact of predictors, e.g., consistency, dysphagia etiology, etc., on observers’ agreement and permits the identification of aspects influencing negatively the level of agreement [11]. By identifying factors that can influence observers’ agreement on measured FEES variables, researchers can better understand the rating process and thereby help develop a procedure to increase observer agreement levels. In that light, the aim of this study is to compare (1) observers’ agreement on FEES measurements in patients with dysphagia of neurological versus head and neck oncological origin, and (2) observers’ behavior in independent versus consensus panel rating.

## Methods

### Subject Selection

Thirty consecutive patients with dysphagia of neurological origin and thirty consecutive patients with dysphagia of head and neck oncological origin were included. All patients underwent FEES examination, from 2010 to 2012, in the Maastricht University Medical Center (MUMC). Oropharyngeal dysphagia was identified by the multidisciplinary team based on clinical assessment and FEES examination. Patients were excluded if they presented severe dyskinesia of the head and neck, suffered from severe mental depression, had cognitive impairment (Mini Mental State Examination score <23), or had concurrent head and neck cancer and a neurological disease.

### Swallowing Assessment

All measurements were performed in the same hospital by the same multidisciplinary team. All subjects underwent the same FEES protocol [12]. During the exam, two consistencies were administered: three 10 cc trials of thin liquid (water dyed with 5 % methylene blue) and three 10 cc trials of thick liquid (applesauce dyed with 5 % methylene blue). All participants were offered the bolus consistencies in the same sequence (thin liquid followed by thick). The tip of the flexible fiberoptic endoscope Pentax FNL-10RP3 (Pentax Canada Inc., Mississauga, Ontario, Canada) was positioned just above the epiglottis. Neither a nasal vasoconstrictor nor a topical anesthetic was administered to the nasal mucosa. Images were obtained using an Alphatron Stroboview ACLS camera, Alphatron Lightsource, and IVACX computerized video archiving system (Alphatron Medical Systems, Rotterdam, the Netherlands) and recorded on a DVD at 30 frames per second.

### Swallowing Measurements

Two students in their last year of medical school without experience in swallowing evaluation were selected as observers. Prior to data collection, they completed an intensive training program on the rating scales of four visuoperceptual ordinal variables (Table 1). The observers were jointly trained in the interpretation of the scales by an expert. A written manual with well-defined descriptions of the levels was available during the training program and the subsequent rating process, and could be consulted anytime. The duration of the training program was pre-determined and consisted of ten training sessions of approximately 1 h each. The training sessions were interspersed with practice periods when the observers had to do test runs separately. Each practice period consisted of 2 h, in average. The results were discussed in the next training session. All FEES exams selected were scored separately by an expert. During the training session, the exams were jointly analyzed and discussed between the observers and the expert. Moreover, observers’ scores of the training session and the practice session were compared to expert scores to assess medical student’s accuracy of FEES interpretation. The training was predominantly targeted to generate sufficient intra- and interobserver agreement levels. After ten training sessions, the statistical analyses of the practice trials showed sufficient interobserver agreement (weighted *κ* ≥ 0.6), so the observers were confident about starting to rate the FEES exams for the present study. All four visuoperceptual ordinal variables were scored for each deglutition. The entire recording of each swallowing act was analyzed at varying speed (slow motion, normal, and frame-by-frame) as often as necessary, using the software program Windows Movie Maker version 5.1 (Microsoft Corporation, Redmond, WA, USA). During training, an equal amount of FEES images were taken from each etiological group for analysis. The observers were blinded to the patients’ medical history and the origin of their oropharyngeal dysphagia. The swallows were scored in random order. Furthermore, observers were advised to limit the duration of the measurement sessions to 2 h to avoid fatigue, which could introduce bias. The process was divided into two separate tasks: independent rating and consensus panel rating. When rating independently, the observers were blinded to each other’s scores; on the consensus panel, the two observers analyzed the swallowing videos together and the scores were determined by consensus agreement. To reach intraobserver agreement, each observer performed repeated measurements independently within a period of 2 weeks. The consensus panel task was also repeated to obtain test–retest agreement. The number of swallows was balanced regarding bolus consistency (thin and thick liquid) and patient group (neurological and oncological origin) for all tasks.

**Table 1:** Description of the ordinal rating scales of the four visuoperceptual FEES variables

FEES variable	Definition	Rating scale
Piecemeal deglutition	Sequential swallowing on the same bolus	0 = one swallow 1 = two swallows 2 = three swallows 3 = four swallows 4 = five or more swallows
Postswallow vallecular pooling	Bolus retention in the valleculae after swallowing	0 = no pooling 1 = filling of less than 50 % of the valleculae 2 = filling of more than 50 % of the valleculae
Postswallow pyriform sinus pooling	Bolus retention in the pyriform sinuses after swallowing	0 = no pooling 1 = trace to moderate pooling 2 = severe pooling up to complete filling of the sinus
Laryngeal penetration/tracheal aspiration	Bolus in the laryngeal vestibule above or on the level of the vocal folds (laryngeal penetration) or bolus passes below the vocal folds (tracheal aspiration)	0 = no laryngeal penetration 1 = laryngeal penetration 2 = tracheal aspiration

### Statistical Analysis

Results were expressed as mean and standard error (SE) for quantitative variables, while frequencies and proportions (%) were used for ordinal variables. The intra- and interobserver agreement was quantified using the linear weighted kappa coefficient. The weighted kappa values were interpreted as poor (0), slight (0.00–0.20), fair (0.21–0.40), moderate (0.41–0.60), substantial (0.61–0.80), and almost perfect agreement (0.81–1) [13]. The standard error of weighted kappa coefficients was adjusted for the repeated measurements taken from the patients [14]. The effect of predictors (dysphagia etiology, different observers, and bolus consistency) on the intra- and interobserver agreement levels and the probability of changing the FEES scores of the independent rating task during the consensus panel were analyzed using a multilevel approach [11]. Random effects relative to the patients were introduced in the models to capture the multiple measurements for each patient (six swallows). The variance of these random effects is denoted by *σ*^2^. Large values indicate heterogeneous agreement levels among patients, while small values indicate homogeneous agreement levels. The intercept is used to give the average agreement levels for a median patient in all the reference categories (i.e., observer 2, thick liquid, neurological patient). A Bayesian approach was used to estimate the parameters in the model. In Bayesian estimation, the prior knowledge about parameters is combined with the observed data to yield a posterior distribution. Vague priors, which express that we do not have prior information on the parameters, were used. The posterior summary measures were obtained using the Markov-Chain Monte Carlo (MCMC) sampling approach. A predictor is said to be significant if the 95 % equal-tailed posterior credibility interval relative to the predictor does not contain the value 0. Data analysis was conducted using R (version 3.0.2 for Windows) and WinBUGS statistical packages.

## Results

### Characteristics of the Subjects

Sixty mentally competent dysphagic patients were included. Thirty had a diagnosis of neurological origin: myotonic dystrophy (14), stroke (4), Parkinson disease (3), amyotrophic lateral sclerosis (2), inclusion body myositis (2), myasthenia gravis (1), Duchenne muscular dystrophy (1), cerebellar syndrome (1), multiple sclerosis (1), and extra-pyramidal syndrome (1). The other thirty had a diagnosis of head and neck oncological origin: laryngeal carcinoma (10), oropharyngeal carcinoma (9), oral cavity carcinoma (5), nasopharyngeal carcinoma (3), hypopharyngeal carcinoma (2), and parotid gland carcinoma (1). All oncological patients completed treatment at least three months prior to the FEES examination, and none of the patients were in a palliative state of care. The mean age in the neurological group was 57 (SE 3.21); in the oncological group it was 65 (SE 2.04). The level of swallowing impairment represented by FEES scores was similar for both groups (Table 2). The exception was the variable laryngeal penetration/tracheal aspiration, for which the oncological group presented significantly higher scores, indicating more severe impairment.

**Table 2:** Frequency distribution of patients per category of the different FEES variables, given as absolute numbers *N* and percentages (%) according to the etiological group

FEES variables	Rating scale	Etiology	
		Oncological	Neurological
Vallecular pooling	0	78 (54)	92 (63)
	1	47 (32)	39 (27)
	2	20 (14)	15 (10)
Pyriform sinus pooling	0	128 (75)	131 (74)
	1	32 (19)	45 (25)
	2	10 (5.9)	1 (0.6)
Piecemeal deglutition	0	26 (15)	39 (22)
	1	59 (34)	74 (43)
	2	36 (21)	32 (18)
	3	14 (8.1)	10 (5.7)
	4	37 (22)	19 (11)
Penetration/aspiration	0	79 (48)	126 (75)
	1	59 (36)	35 (21)
	2	27 (16)	7 (4.2)

The scores of the observer with the highest intraobserver agreement level were used for the analysis

### Number of Swallows Analyzed

In total, 360 swallows were recorded (six swallows per patient). Two observers scored all 360 independently within a period of 3 months. From these, 120 swallows were randomly selected and scored by both observers also in a consensus panel setting within a period of 3 weeks. To investigate intraobserver agreement, the two observers independently repeated the measurement of 80 randomized swallows within a period of 2 weeks. For the test–retest agreement of the consensus panel, the observers repeated the measurement of 40 randomized swallows within a period of 1 week.

### Intraobserver Agreement

The level of intraobserver agreement ranged from 0.79 to 0.93 for observer 1 and from 0.76 to 0.90 for observer 2 (Table 3). The posterior distribution of the Bayesian non-linear mixed model parameters for intraobserver agreement is summarized in Table 4. The level of intraobserver agreement was similar for both observers, with the exception of postswallow vallecular pooling: observer 1 had a higher intraobserver agreement than observer 2 on that variable. There was no difference in intraobserver agreement between oncological and neurological patients, nor between thin and thick liquid consistencies.

**Table 3:** Linear weighted kappa coefficient (SE) of agreement for all rating tasks

FEES variables	Intraobserver agreement		Interobserver agreement			Intrapanel agreement
	Observer 1	Observer 2	Thin liquid	Thick liquid	Total	Total
Piecemeal deglutition	0.86 (0.041)	0.90 (0.026)	0.84 (0.033)	0.93 (0.019)	0.88 (0.020)	0.95 (0.029)
Postswallow vallecular pooling	0.93 (0.041)	0.79 (0.068)	0.30 (0.075)	0.76 (0.040)	0.65 (0.037)	0.85 (0.071)
Postswallow pyriform sinus pooling	0.79 (0.054)	0.76 (0.084)	0.55 (0.071)	0.67 (0.069)	0.61 (0.059)	0.91 (0.068)
Laryngeal penetration/tracheal aspiration	0.79 (0.064)	0.79 (0.066)	0.82 (0.037)	0.58 (0.070)	0.73 (0.035)	0.93 (0.049)

*SE* standard error

**Table 4:** Posterior distribution [mean (SD) and 95 % equal-tailed credibility interval (CI)] of the parameters of the Bayesian non-linear mixed model for intraobserver agreement

	Piecemeal deglutition			Postswallow vallecular pooling			Postswallow pyriform sinus pooling			Laryngeal penetration/tracheal aspiration		
	Mean (SD)	95 % CI		Mean (SD)	95 % CI		Mean (SD)	95 % CI		Mean (SD)	95 % CI	
Intercept	1.45 (0.39)	0.81	2.24	−0.39 (0.65)	−1.57	0.88	0.93 (0.42)	0.24	1.95	0.75 (0.34)	0.029	1.41
Observer^a^	0.12 (0.19)	−0.16	0.63	1.19 (0.61)	0.016	2.34	−0.067 (0.36)	−0.85	0.60	−0.056 (0.27)	−0.61	0.46
Consistency^b^	−0.14 (0.25)	−0.66	0.34	0.80 (0.61)	−0.43	1.93	−0.39 (0.30)	−1.02	0.18	0.27 (0.30)	−0.27	0.86
Group^c^	−0.23 (0.26)	−0.83	0.23	0.053 (0.51)	−1.16	0.95	0.22 (0.45)	−0.71	1.02	−0.15 (0.34)	−0.86	0.48
*σ* ^2^	0.60 (0.60)	0.0024	1.98	0.14 (0.24)	0.00	0.79	0.14 (0.24)	0.00	0.79	0.19 (0.17)	0.00	0.59

To facilitate interpretation of the table, a more detailed description is given. Mean, SD, and 95 % CI are presented separately per FEES variable. When ‘0’ is not entailed in the 95 % CI, the difference between the predictors (observer 1 and observer 2, or thin and thick liquid consistency, or neurological and oncological group) is statistically significant. A positive mean indicates that the agreement of the predictor used as reference is lower. For instance, in the line ‘observer,’ the intraobserver agreement level between the two observers is compared. In the 95 % CI column for the variable postswallow vallecular pooling, ‘0’ is not entailed (0.016, 2.34). It means that a statistically significant difference was found in the intraobserver agreement level between the two observers when rating this variable. As observer 2 is used as a reference, a positive mean (1.19) indicates that intraobserver agreement for observer 2 was lower than that for observer 1 when rating postswallow vallecular pooling

*SD* standard deviation

The groups used as a reference are:

^a^Observer 2 for observer effect

^b^Thick liquid for bolus consistency

^c^Neurological patients for the etiological group

### Interobserver Agreement

Interobserver agreement levels are presented in Table 3 according to the bolus consistency. The posterior distribution of the Bayesian non-linear mixed model parameters for interobserver agreement is summarized in Table 5. Interobserver agreement was lower for thin liquid than for thick liquid swallow trials on the variables piecemeal deglutition and postswallow vallecular pooling. The opposite was observed for the measurements of the variable laryngeal penetration/tracheal aspiration. Interobserver agreement was slightly lower on the postswallow pyriform sinus pooling scale for thin liquid trials compared with thick liquid ones. On closer inspection, disagreement between the two observers occurred mainly at the first two levels of the scale (normal and mild impairment). There was no difference in the level of interobserver agreement for oncological versus neurological patients.

**Table 5:** Posterior distribution [mean (SD) and 95 % equal-tailed credibility interval (CI)] of the parameters of the Bayesian non-linear mixed model for interobserver agreement

	Piecemeal deglutition			Postswallow vallecular pooling			Postswallow pyriform sinus pooling			Laryngeal penetration/tracheal aspiration		
	Mean (SD)	95 % CI		Mean (SD)	95 % CI		Mean (SD)	95 % CI		Mean (SD)	95 % CI	
Intercept	1.45 (0.21)	1.08	1.88	0.70 (0.15)	0.41	1.01	0.43 (0.19)	0.072	0.80	0.36 (0.16)	0.056	0.67
Consistency^a^	−0.51 (0.17)	−0.86	−0.19	−0.86 (0.20)	−1.27	−0.51	−0.28 (0.18)	−0.66	0.081	0.64 (0.17)	0.33	0.98
Group^b^	−0.14 (0.20)	−0.53	0.25	−0.0011 (0.19)	−0.37	0.36	−0.049 (0.21)	−0.48	0.34	0.012 (0.17)	−0.33	0.32
*σ* ^2^	0.18 (0.13)	0.0045	0.48	0.051 (0.060)	0.00	0.21	0.13 (0.086)	0.0012	0.32	0.022 (0.017)	0.00	0.063

To facilitate interpretation of the table, a more detailed description is given. Mean, SD, and, 95 % CI are presented separately per FEES variable. When ‘0’ is not entailed in the 95 % CI, the difference between the predictors (thin and thick liquid consistency, or neurological and oncological group) is statistically significant. A positive mean indicates that the agreement of the predictor used as reference is lower. For instance, in the line consistency, the agreement level between thin and thick liquid consistencies is compared. ‘0’ is not entailed in the 95 % CI of all FEES variables, except for postswallow pyriform sinus pooling (−0.66, 0.081). It means that there is a statistically significant difference on the agreement level depending on the consistency scored. A negative mean for piecemeal deglutition (−0.51) and postswallow vallecular pooling (−0.86) indicates that the interobserver agreement for thick was higher than that for thin liquid

*SD* standard deviation

The groups used as reference are:

^a^Thick liquid for bolus consistency

^b^Neurological patients for the etiological group

### Consensus Panel Agreement

The intrapanel agreement level is presented in Table 3. Comparison of the scores given independently to those given on the consensus panel for exactly the same FEES measurement reveals that the magnitude of the changes in the score varies according to the FEES variable assessed. The probability that an independent score would change on the consensus panel was 27 % for postswallow vallecular pooling, 17 % for postswallow pyriform sinus pooling, 16 % for piecemeal deglutition, and 14 % for laryngeal penetration/tracheal aspiration. The frequency of such changes was slightly higher for the variable postswallow vallecular pooling during thick liquid swallows compared with thin liquid ones (Table 6). No statistically significant difference was detected in the frequency of changes in FEES measurements between etiological groups and between observers, with one exception: postswallow pyriform sinus pooling, where changes were more frequent for observer 1.

**Table 6:** Posterior distribution [mean (SD) and 95 % equal-tailed credibility interval (CI)] of the parameters of the Bayesian multilevel probit model for the probability of changing the ordinal FEES scores of the independent rating task during the consensus panel rating task

	Piecemeal deglutition			Postswallow vallecular pooling			Postswallow pyriform sinus pooling			Laryngeal penetration/tracheal aspiration		
	Mean (SD)	95 % CI		Mean (SD)	95 % CI		Mean (SD)	95 % CI		Mean (SD)	95 % CI	
Intercept	−1.09 (0.46)	−2.04	−0.22	−0.77 (0.24)	−1.26	−0.30	−0.62 (0.29)	−1.21	−0.069	−0.70 (0.25)	−1.19	−0.21
Observer^a^	0.20 (0.23)	−0.24	0.64	−0.13 (0.21)	−0.54	0.28	0.73 (0.24)	0.27	1.20	0.10 (0.21)	−0.30	0.52
Consistency^b^	0.034 (0.23)	−0.41	0.48	−0.38 (0.21)	−0.79	0.025	0.15 (0.22)	−0.29	0.59	0.30 (0.21)	−0.10	0.71
Group^c^	0.12 (0.60)	−1.07	1.32	0.15 (0.27)	−0.38	0.68	0.21 (0.37)	−0.50	0.96	0.17 (0.29)	−0.39	0.76
*σ*²	1.38 (1.14)	0.33	4.05	0.16 (0.15)	0.00	0.53	0.37 (0.31)	0.036	1.18	0.20 (0.21)	0.002	0.72

To facilitate interpretation of the table, a more detailed description is given. Mean, SD, and 95 % CI are presented separately per FEES variable. When ‘0’ is not entailed in the 95 % CI, the difference between the predictors (observer 1 and observer 2, or thin and thick liquid consistency, or neurological and oncological group) is statistically significant. A positive mean indicates that the agreement of the predictor used as reference is lower. For instance, in the line ‘Observers,’ the comparison between the observers’ probability of changing FEES scores of the independent rating task during the consensus panel rating task is analyzed. ‘0’ is entailed in the 95 % CI of all FEES variables, except for postswallow pyriform sinus pooling (0.27, 1.20). It means that a statistically significant difference was found in the observers’ probability of changing the FEES scores of the independent rating task during the consensus panel rating task when rating this variable. The positive mean (0.73) indicates that observer 1 changed the scores more frequently than observer 2 during the panel task

*SD* standard deviation

The groups used as reference are:

^a^Observer 2 for observer effect

^b^Thick liquid for bolus consistency

^c^Neurological patients for the etiological group

## Discussion

The two main aspects of an outcome measurement are *validity* (how accurate are the measurements) and *reproducibility* (how similar are the results of the repeated measurements). Although both concepts are related, they can be investigated separately. Observers’ agreement is the first step to show validity as it is not possible to have a valid scale if the measurements are not reproducible. The term *reproducibility* can be used to comprise two concepts, agreement and reliability, because both concepts concern the question of whether measurement results are reproducible in test–retest situations. Agreement parameters assess how close the results of the repeated measurements are, by estimating the measurement error in repeated measurements. Reliability parameters assess whether study objects, often persons, can be distinguished from each other despite measurement errors. In that case, the measurement error is related to the variability between persons. Consequently, reliability parameters are highly dependent on the heterogeneity of the study sample, while the agreement parameters, based on measurement error, are more a pure characteristic of the measurement instrument [15]. Therefore, the present study analyzes intra- and interobserver agreement and explores any discrepancy in the ratings to better understand the causes of disagreement among observers. The effects of dysphagia etiology, different observers, and bolus consistency on the agreement levels were analyzed in two types of rating tasks: independent rating (intra- and interobserver agreement) and consensus panel rating (intrapanel observer agreement).

The effect of dysphagia etiology (neurological or head and neck oncological origin) on the agreement levels was also analyzed in all rating tasks. Except for aspiration where oncological patients presented higher scores, there was no effect of the dysphasia etiology on the other FEES variables. The absence of an effect of dysphagia etiology on agreement was unexpected, as it was presumed that alterations in the anatomy and physiology of the pharynx and/or larynx, secondary to cancer treatment, would influence the observers’ agreement on the ratings. Apparently, the selected FEES variables are appropriate to evaluate both etiological groups. The results suggest that the training program offered sufficient information to enable the observers to evaluate swallowing function using FEES without taking changes in the anatomy and physiology of swallowing into account.

In the independent rating task, the intraobserver agreement level was similar for both observers, and there was no effect of bolus consistency. These findings show that the two observers had a similar interpretation of the ordinal scoring system and were consistent when repeating the measurements. In accordance with previous studies, intraobserver agreement was higher than the agreement between the two observers (interobserver agreement) [9, 12].

Overall, interobserver agreement levels were substantial (*κ* > 0.61). However, a more detailed analysis demonstrated that agreement levels were affected by bolus consistency. For instance, during thin liquid trials, interobserver agreement for postswallow vallecular and pyriform sinus pooling was fair to moderate (0.30 and 0.55, respectively). The lower interobserver agreement recorded for these measured variables concurs with findings reported elsewhere [12]. Although bolus consistency is known to influence swallowing performance, the impact of consistency on observer agreement is underexplored [16, 17].

The lower levels of interobserver agreement might be explained as follows. First, even though the observers understood the ordinal scoring system well, as confirmed by the intraobserver agreement levels, they did not reach consensus on the cut-off points. The description of the rating scale does not give the precise range of each ordinal level, which leaves it up to the observers to set their own boundaries. Second, as thin liquid consistency is less cohesive, the bolus is not concentrated but instead spreads in the valleculae or pyriform sinus, thereby hindering an estimation of the amount of pooling. Moreover, the very nature of the FEES images makes it difficult to quantify precisely the amount of bolus left after swallowing [9, 18].

The intrapanel observer agreement levels were slightly higher than the intraobserver levels on the independent rating task. That difference suggests that consensus panel rating might offer an alternative to independent rating of FEES exams, as the discussion of cases in a panel may improve concordance [19]. However, the agreement level obtained between two separate consensus panels with different members still needs to be explored, particularly in comparison to individual interobserver agreement levels.

Observers were consistent when re-scoring swallows independently or on the consensus panel. However, when repeating the task on the panel, they frequently adjusted the scores they had given previously when rating exactly the same measurements independently. That tendency to change in a panel setting reflects the observers’ individual interpretation of the ordinal FEES scoring system. Furthermore, the probability of changing scores during the consensus panel rating task was similar for both observers. One explanation might be that, besides being inexperienced in rating FEES exams, the observers had followed the same intensive training program. Consensus panel ratings performed by observers with different levels of experience, or without specific training on FEES measurements, might yield other results.

### Limitations of the Study

The present study was based on FEES ratings of two observers. Comparing scores by a larger number of observers might produce different results. Furthermore, including students without experience in swallowing evaluation was a pre-experimental choice because we were interested in the agreement between naïve observers. Including more experienced observers might produce different results. The ordinal scales of the FEES outcome variables have been described in several previously published studies [12, 17]. However, they were not validated yet, which might have implications for the interpretation of the results.

## Conclusion

Observers’ agreement on FEES measurements was influenced by bolus consistency and not by dysphagia etiology, as defined in the present study design. It would be preferable to analyze observer agreement on FEES measurements according to bolus consistency, as this variable apparently affects the interpretation of the outcome. This study illustrates how the identification of factors that might influence agreement levels could elucidate the rating process. Investigations such as this could assist in developing a more precise measurement scale to improve observer agreement on measurements in FEES exams.
